# Deformation of Soft Tissue and Force Feedback Using the Smoothed Particle Hydrodynamics

**DOI:** 10.1155/2015/598415

**Published:** 2015-08-31

**Authors:** Xuemei Liu, Ruiyi Wang, Yunhua Li, Dongdong Song

**Affiliations:** ^1^North China University of Water Resource and Electric Power, Zhengzhou 450011, China; ^2^School of Automation Science and Electrical Engineering, Beihang University, Beijing 100191, China; ^3^Henan Radio & Television University, Zhengzhou 450011, China

## Abstract

We study the deformation and haptic feedback of soft tissue in virtual surgery based on a liver model by using a force feedback device named PHANTOM OMNI developed by SensAble Company in USA. Although a significant amount of research efforts have been dedicated to simulating the behaviors of soft tissue and implementing force feedback, it is still a challenging problem. This paper introduces a kind of meshfree method for deformation simulation of soft tissue and force computation based on viscoelastic mechanical model and smoothed particle hydrodynamics (SPH). Firstly, viscoelastic model can present the mechanical characteristics of soft tissue which greatly promotes the realism. Secondly, SPH has features of meshless technique and self-adaption, which supply higher precision than methods based on meshes for force feedback computation. Finally, a SPH method based on dynamic interaction area is proposed to improve the real time performance of simulation. The results reveal that SPH methodology is suitable for simulating soft tissue deformation and force feedback calculation, and SPH based on dynamic local interaction area has a higher computational efficiency significantly compared with usual SPH. Our algorithm has a bright prospect in the area of virtual surgery.

## 1. Introduction

Virtual surgery simulation is an important application of virtual reality aiming at establishing vivid virtual surgery environment with all kinds of medical image data so that doctors and trainees can make use of it to do surgical trainings.

Traditionally, the animal model and corpses are considered the most common training model. However, the animal model has fundamental differences in anatomy and tissue consistency compared to human tissue, and the corpses are expensive and cannot be reused. Therefore, it is difficult to reach a proficient skill level for surgeons.

This calls for a more innovative training system for surgical residents. Virtual surgery simulation system can meet it. In such a system, the surgeons would be able to interact with the virtual three-dimensional models of organs using their sense of vision and touch.

In virtual surgery simulation, the deformation model of soft tissue is the decisive factor of visual effect and accuracy of force feedback and has been widely studied in the computer graphics and computer aided design communities [1]. Currently there are two most widely used physical models of soft tissue. One is Finite Element Models (FEM) [1–5] and the other one is Mass-Spring (M-S) Models [6–9].

A major advantage of FEM is that it uses continuum mechanics and has a solid mathematical foundation. Another advantage is that FEM requires only a few material parameters to describe a physical system's response. However, there are main issues existing in this approach, the first one is the heavy computational load which cannot ensure real-time and the second one is the impact of cutting on the precomputed response. Compared with FEM, M-S does not need parameters of the continuum, so it is easier to implement and handle topological changes at a reduced computational cost. The computation is specifically efficient. However, mass-spring systems are not necessarily accurate which are not built upon elasticity theory. Primarily, most of such systems are not convergent; next, the parameters of every mass and spring in this model cannot be obtained in the measurement but can often be chosen arbitrarily. Therefore, M-S has low precision and poor stability. The common methods such as M-S and FEM are all built on meshes. Malformation and distortion could arise and they cannot ideally satisfy the needs of real-time virtual surgery simulation and the interaction and attachment between surgery tools and soft tissue must have effect on some specific points when using methods built on meshes. Moreover, we must remesh frequently because of topology changes in cutting and suturing simulations, which leads to expensive computation and bad haptic performance.

In order to overcome these problems, certain meshes should be avoid being used. De et al. [10] developed a point collocation-based method of finite spheres technique to simulate the interaction of surgical tool and soft tissue. Jansson and Vergeest [11] developed a discrete mechanics model for deformation bodies, incorporating behavior such as motion, collision, and deformation. Müller et al. [12] developed a shape matching method for meshless deformations. The meshfree technique is no mesh dependence, strongly self-adaptive, and smoothly continuous. Moreover, it can describe continuum biomechanical characteristics of soft tissue. Meshless method has a broad application prospect in the virtual surgery simulation.

In this paper, we present an application of smoothed particle hydrodynamics (SPH) method to simulate the interaction between virtual surgical tool and soft tissue which is a meshfree technique. In SPH the continuum properties are discredited on smooth particles, the stress-strain governing equations are formulated in a Lagrange frame, and the derivatives are computed by taking the derivatives of the particle kernels. The SPH method can deal with large deformation and be capable of resolving problems with large deformations for both solids and fluids.

In Section 2, we introduce the viscoelastic model used in this paper. In Section 3, we provide a brief introduction to the SPH method. In Section 4, we develop a SPH method based on dynamic local interaction area of performing real time deformation. Finally, in Section 5 simulation results are presented.

## 2. Voigt Viscoelastic Model

The virtual surgery simulation focuses on requirement of (1) reality; (2) real time performance; (3) performance of quantitative deformation; (4) easy calculation for generating the values for force feedback. Requirement (1) depends on the accuracy of the underlying biomechanics models. According to biomechanics literature [9], soft biological tissues exhibit complex viscoelastic in nature. Currently, most of the existing deformation methods are built on an elastic mechanical model, which cannot describe the deformation behavior of soft tissue precisely.

Here, we simulate the biological tissue as a linear viscoelastic solid and use Voigt model to establish the relationship between stress and strain. According to Voigt model, stress tensor of isotropy material can be divided into spherical tensor and deviator tensor, and strain tensor can be divided into volume deformation and distortion of same volume:

(1) $$\begin{array}{c} \sigma_{\alpha \beta }=S_{\alpha \beta }+\frac{\delta_{\alpha \beta }\sigma_{kk}}{3}, \end{array}$$

(2) $$\begin{array}{c} \varepsilon_{\alpha \beta }=e_{\alpha \beta }+\frac{\delta_{\alpha \beta }\varepsilon_{kk}}{3}, \end{array}$$

where *α*, *β* = *x*, *y*, *z*, *S*_*αβ*_, *σ*_*kk*_ are the components of the partial stress tensor and spherical stress tensors, respectively, *e*_*αβ*_, *ε*_*kk*_ are the components of the partial strain tensor and spherical strain tensors, respectively, and *δ*_*αβ*_ is the Kronecker symbol.

According to Voigt model, 3D viscoelastic constitutive equations can be described as follows:

(3) $$\begin{array}{c} S_{\alpha \beta }=E\cdot e_{\alpha \beta }+\eta \cdot \frac{de_{\alpha \beta }}{dt},\\ \sigma_{kk}=E\cdot \varepsilon_{kk}+\eta \cdot \frac{d\varepsilon_{kk}}{dt}. \end{array}$$

Then we establish strain-displacement geometric equation as follows:

(4) $$\begin{array}{c} \varepsilon_{xx}=\frac{\partial u}{\partial x},\;\;\varepsilon_{xy}=\frac{\partial u}{\partial y}+\frac{\partial v}{\partial x}\\ \varepsilon_{yy}=\frac{\partial v}{\partial y},\;\;\varepsilon_{yz}=\frac{\partial v}{\partial z}+\frac{\partial w}{\partial y}\\ \varepsilon_{zz}=\frac{\partial w}{\partial z},\;\;\varepsilon_{zx}=\frac{\partial w}{\partial x}+\frac{\partial u}{\partial z}, \end{array}$$

where *u*, *v*, *w* are the components of displacement along the coordinate axis, *ε*_*xx*_, *ε*_*yy*_, *ε*_*zz*_ are the components of normal strain, and *ε*_*xy*_, *ε*_*yz*_, *ε*_*zx*_ are the components of shear strain.

## 3. The Basic Theory of SPH

### 3.1. SPH Formulation

SPH method was firstly put forward by Lucy [13]. It is featured by Lagrangian and particle properties, fully meshless and explicit in coping with those trouble-making nonlinear problems [14]. SPH has been applied to model a wide range of problems [15, 16] such as free surface flows [17, 18], viscous flows [19], and multiphase flows [20].

At the heart of the SPH method is an interpolation technique for evaluating arbitrary field functions. To illustrate this, consider a field function *f*(*x*) in an unbounded domain. The value of the function at a point *x* can be obtained by a convolution with the Dirac delta distribution function *δ*:

(5) $$\begin{array}{c} f\left(x\right)=\overset{}{\underset{\Omega }{\int }}f\left(y\right)\delta \left(x-y\right)dy. \end{array}$$

Dirac delta function *δ* has the following property:

(6) $$\begin{array}{c} \delta \left(x-y\right)=\left\{\begin{array}{cc} 1, & x=y\\ 0, & x\neq y. \end{array}\right. \end{array}$$

In SPH, the singular Dirac delta function is approximated by a smooth kernel function *W* to yield an approximation. The classical SPH method employs a finite number of particles to discretize the continuum. Each particle located at the position vector *x* carries field variables such as mass and density and moves with the material velocity. Therefore, (5) can be transformed to the superposition sum of discretization form of all particles support in the domain:

(7) $$\begin{array}{c} f\left(x\right)=\overset{N}{\underset{j=1}{\sum }}m_{j}\frac{f\left(y\right)}{\rho_{j}}W\left(x-y,h\right), \end{array}$$

where *m*_*j*_, *ρ*_*j*_, *y* are the mass, density, and position of particle *j*, respectively, and *h* is the smoothing length.

Correspondingly, the derivative of function *f*(*x*) can be introduced in the following form:

(8) $$\begin{array}{c} \nabla f\left(x\right)=-\overset{N}{\underset{j=1}{\sum }}\frac{m_{j}}{\rho_{j}}f\left(y\right)\cdot \nabla W\left(x-y,h\right)dy. \end{array}$$

Smooth kernel function strongly affects the stability and accuracy of SPH and plays a very important role. In this paper, the kernel function used in the present investigation is the most widely used cubic spline function [21]:

(9) $$\begin{array}{c} W\left(R,h\right)=\frac{3}{2\pi h^{3}}\times \left\{\begin{array}{cc} \frac{2}{3}-R^{2}+\left(\frac{1}{2}\right)R^{3}, & 0\leq R<1\\ \left(\frac{1}{6}\right){\left(2-R\right)}^{3}, & 1\leq R<2\\ 0, & R\geq 2, \end{array}\right. \end{array}$$

where *R* denotes the relative distance between the *i*th and the *j*th particle at the position of *x*_*i*_, *x*_*j*_, *R* = *r*/*h* = |*x*_*i*_ − *x*_*j*_|/*h*, *r* is the distance of two particles, and *h* is the smoothing length. The derivative of the kernel function is as follow:

(10) $$\begin{array}{c} \frac{\partial W_{ij}}{\partial x^{\beta }}=\frac{x_{i}^{\beta }-x_{j}^{\beta }}{u}\cdot \frac{1}{\pi h^{4}}\times \left\{\begin{array}{cc} -3R+1.25R^{2}, & 0\leq R<1\\ -0.75{\left(2-R\right)}^{2}, & 1\leq R<2\\ 0, & R\geq 2. \end{array}\right. \end{array}$$

### 3.2. Search for the Neighbor Particles

**Figure 1:**
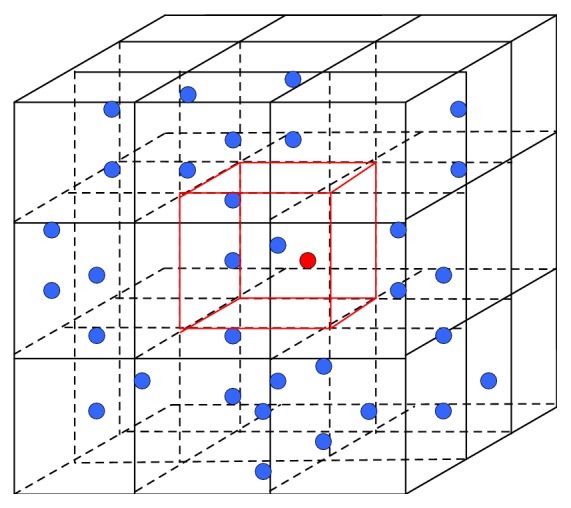
3D grid of cells for searching neighbor particles.

In order to make the experiment simpler, we set the smoothing length in this paper be a constant, so using the chain table search method is very effective. The basic idea is laying a temporary grid in the problem domain, as shown in Figure 1; the grid cell size is consistent with the size of support domain spatial, when searching particles, for a given particle *i*, the neighbor particle only in the same grid or closely neighbor grid. This search method limited the search scope to around the center grid cell so that time is short and search efficiency is high.

### 3.3. Momentum Equation of SPH

In SPH, movement of particles follows the law of conservation of momentum, and any particle conforms to the momentum equation as follows:

(11) $$\begin{array}{c} \frac{dv^{\alpha }}{dt}=\frac{1}{\rho }\frac{\partial \sigma^{\alpha \beta }}{\partial x^{\beta }}, \end{array}$$

where *v* is the velocity vector, *t* is time, *ρ* is the density of particle, *σ* is stress of particle, and *x* is coordinate vector.

Equation (11) can be transformed as follows using particle approximation method:

(12) $$\begin{array}{c} \frac{dv_{i}^{\alpha }}{dt}=\overset{N}{\underset{j=1}{\sum }}m_{j}\frac{\sigma_{i}^{\alpha \beta }+\sigma_{j}^{\alpha \beta }}{\rho_{i}\rho_{j}}\frac{\partial W_{ij}}{\partial x_{i}^{\beta }}. \end{array}$$

Consider the following equation:

(13) $$\begin{array}{c} \frac{1}{\rho }\frac{\partial \sigma^{\alpha \beta }}{\partial x^{\beta }}=\frac{\partial }{\partial x^{\beta }}\left(\frac{\sigma^{\alpha \beta }}{\rho }\right)+\frac{\sigma^{\alpha \beta }}{\rho^{2}}\frac{\partial \rho }{\partial x^{\beta }}. \end{array}$$

Then (12) can be written as

(14) $$\begin{array}{c} \frac{dv_{i}^{\alpha }}{dt}=\overset{N}{\underset{j=1}{\sum }}m_{j}\left(\frac{\sigma_{i}^{\alpha \beta }}{\rho_{i}^{2}}+\frac{\sigma_{j}^{\alpha \beta }}{\rho_{j}^{2}}\right)\frac{\partial W_{ij}}{\partial x_{i}^{\beta }}. \end{array}$$

Here, the density of each particle can be computed as

(15) $$\begin{array}{c} \rho_{i}=\overset{N}{\underset{j=1}{\sum }}m_{j}W_{ij}. \end{array}$$

Finally, we convert (4) with SPH:

(16) $$\begin{array}{c} \varepsilon_{i}^{xx}=\frac{\partial u_{i}}{\partial x}=\overset{N}{\underset{j=1}{\sum }}\frac{m_{j}}{\rho_{j}}\cdot u_{j}\cdot \frac{\partial W_{ij}}{\partial x},\\ \varepsilon_{i}^{yy}=\frac{\partial v_{i}}{\partial y}=\overset{N}{\underset{j=1}{\sum }}\frac{m_{j}}{\rho_{j}}\cdot v_{j}\cdot \frac{\partial W_{ij}}{\partial y},\\ \varepsilon_{i}^{zz}=\frac{\partial w_{i}}{\partial z}=\overset{N}{\underset{j=1}{\sum }}\frac{m_{j}}{\rho_{j}}\cdot w_{j}\cdot \frac{\partial W_{ij}}{\partial z},\\ \varepsilon_{i}^{xy}=\frac{\partial u_{i}}{\partial y}+\frac{\partial v_{i}}{\partial x}=\overset{N}{\underset{j=1}{\sum }}\frac{m_{j}}{\rho_{j}}\cdot u_{j}\cdot \frac{\partial W_{ij}}{\partial y}+\overset{N}{\underset{j=1}{\sum }}\frac{m_{j}}{\rho_{j}}\cdot v_{j}\cdot \frac{\partial W_{ij}}{\partial x},\\ \varepsilon_{i}^{yz}=\frac{\partial v_{i}}{\partial z}+\frac{\partial w_{i}}{\partial y}=\overset{N}{\underset{j=1}{\sum }}\frac{m_{j}}{\rho_{j}}\cdot v_{j}\cdot \frac{\partial W_{ij}}{\partial z}+\overset{N}{\underset{j=1}{\sum }}\frac{m_{j}}{\rho_{j}}\cdot w_{j}\cdot \frac{\partial W_{ij}}{\partial y},\\ \varepsilon_{i}^{zx}=\frac{\partial w_{i}}{\partial x}+\frac{\partial u_{i}}{\partial z}=\overset{N}{\underset{j=1}{\sum }}\frac{m_{j}}{\rho_{j}}\cdot w_{j}\cdot \frac{\partial W_{ij}}{\partial x}+\overset{N}{\underset{j=1}{\sum }}\frac{m_{j}}{\rho_{j}}\cdot u_{j}\cdot \frac{\partial W_{ij}}{\partial z}. \end{array}$$

## 4. SPH Method Based on Dynamic Local Interaction Area

### 4.1. Fundamental

SPH is computationally costly. When the number of particles in the model becomes larger, the computing will be multiplied, and real-time performance will be poor with no steady and continuous feedback force output. In order to improve the computational efficiency, we consider that when the node receives external force from the surgical instruments, the internal force will be decaying gradually with the increase of transmission distance and be just a smaller propagation beyond a certain distance, which can be neglected according to Saint-Venant principle. Therefore, we propose a SPH method based on dynamic local interaction area to perform the deformation simulation and the calculation of force feedback, which maintains high touch frame and improves the accuracy of haptic interaction at the same time.

The purpose of employing the SPH based on dynamic local interaction area to compute the deformation process is improving the computational efficiency by reducing the number of particles participating in the computation, and its basic idea is to divide the model into deformation area and nondeformation area and assume that the deformation is only affected by nodes in the deformation area, and the nodes in nondeformation area will remain still. Deformation area contains two parts, namely, interaction area and transmission area. Interaction area is the region greatly influenced by the surgical instruments, in which the SPH method is adopted to do accurate calculation of particles; transmission area is the effective propagation range of force, in which the physical quantities of all particles are obtained by decaying physical quantities of the force points.

**Figure 2:**
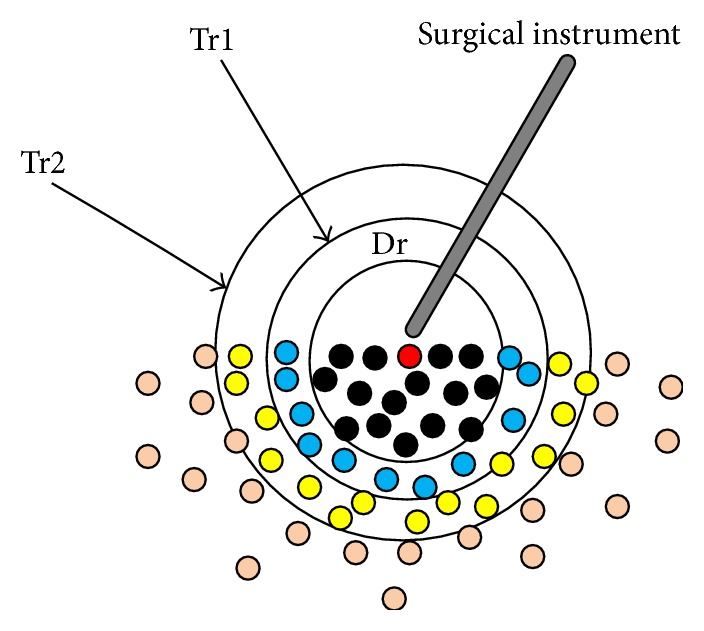
Schematic of SPH based on dynamic local interaction area.

In this paper, the transmission number is set to be two.  Figure 2 illustrates the conceptual schematic of SPH based on dynamic local interaction area.

The surgical instrument contacted the model by the red point at the contact point. Define the Dr as interaction area radius and Tr1, Tr2 as transmission radius, respectively. Once the contact is detected, the algorithm calculates the relative distance between the force point and other points of the model. If the distance is less than Dr, we incorporate the points into the computation model and render the deformation, using SPH method to solve the motion equations and calculate the stress and strain. If the distance is between Dr and Tr1, to get the acceleration of particles, we multiply the force point acceleration by a weighting factor directly and then calculate the position vectors in terms of Newton's Second Law. Calculate the velocity and position vector of the particles in other transmission layers in the same way until the acceleration becomes zero. At this point, the weighting factor is zero, implying that the particles are located in the nondeformation area and will not be calculated any more.

### 4.2. Determination of the Interaction Area

The interaction area is determined by two factors: the force point and the applied load. Force point determines the location of interaction area, and the load applied on the model influences the scope of force.

#### 4.2.1. Determine the Location of Interaction Area Dynamically according to the Force Point

**Figure 3:**
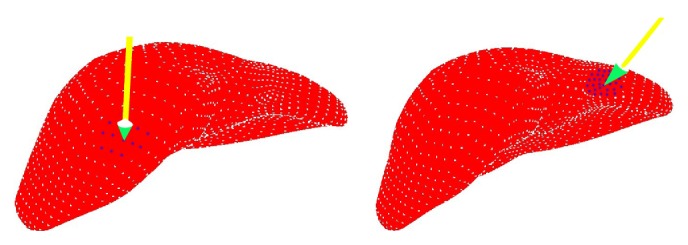
Dynamic division of interaction area.

We manipulate the force feedback device to roam in the virtual environment and apply the force to any point of the model, so the interaction area can be determined dynamically depending on the force point location, as shown in Figure 3.

#### 4.2.2. Determine the Scope of the Interaction Area Dynamically according to the Load

In the SPH method, the influence domain of any particle is associated with its smooth radius *h*; therefore, we define interaction area radius Dr to be a linear function of *h*. Once being defined, Dr will remain constant throughout the computation. Similarly, we also define transmission radius Tri to be a linear function of *h*, where *i* indicates the *i*th level of propagation. The computation formulas of Dr and Tri are as follows:

(17) $$\begin{array}{c} Dr=k_{1}h,\\ Tri=k_{1}h+k_{2}h. \end{array}$$

When an external force *f*_ext_ is applied to the model, the larger *f*_ext_ is, the wider influence range will become, so the greater interaction area and transmission area are. Therefore, the parameter *k*_1_ in (17) is related to *f*_ext_. We define *k*_1_ as follows:

(18) $$\begin{array}{c} k_{1}=\frac{f_{ext}}{2}. \end{array}$$

So (17) can be converted as

(19) $$\begin{array}{c} Dr=\frac{f_{ext}}{2}h\\ Tri=\frac{f_{ext}}{2}h+k_{2}h. \end{array}$$

Here, the parameter *k*_2_ can be computed as

(20) $$\begin{array}{c} k_{2}=\frac{1}{2}i, \end{array}$$

where *i* indicates the *i*th level of propagation.

Equation (17) can divide the model into interaction area and influence area dynamically according to the load. So the algorithm is self-adaptive.

**Figure 4:**
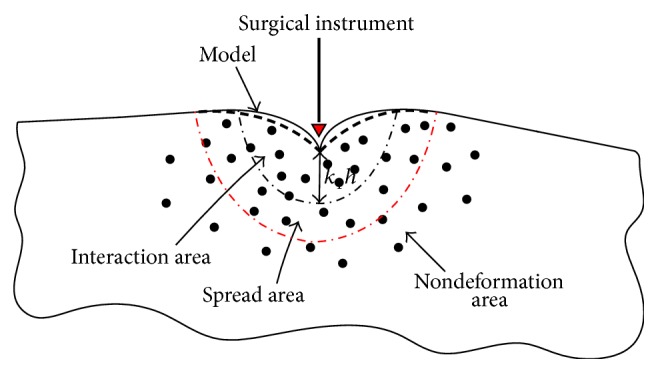
The division for deformation area and nondeformation area.

The division of deformation area and nondeformation is shown in Figure 4.

### 4.3. Weighting Factor

When the model is on load applied by the surgical instrument, the internal force will decrease with the increase of propagation. But the propagation will no longer increases when the force spreads beyond a certain distance. Therefore, we can set different transmission layers to improve the simulation accuracy.

The particles in different transmission layers have different effect on the deformation of the model. So we set weighting factors to indicate the influence degrees of different transmission layers. The farer transmission layer is, the smaller weighting vector is, meaning that the particles in this layer have less contribution to the deformation.

Assuming that the transmission layer is *N*, so the force in (*N* + 1)th layer decays to zero. Therefore, the computation formula of weighting vector is

(21) $$\begin{array}{c} w\left(i\right)=\frac{N+1-i}{N+1}, \end{array}$$

where *i* indicates the *i*th level of propagation. In this paper, we set *N* = 2, so the weighting vector can be computed as (3 − *i*)/3.

### 4.4. Collision Detection

Collision detection is one of the most important issues in developing a multimodal surgery simulation. It is the prerequisite of soft tissue deformation calculation; fast and accurate collision detection algorithm directly affects the authenticity of the human-computer interaction, in which one needs to ensure that there has indeed been a contact between the surgical instrument and the model in virtual scene. Most collision detection algorithms approximate the models in the scene using bounding volumes such as axis aligned bounding boxes (AABBs) [22–24], oriented bounding boxes (OBBs) [25, 26], spheres [27, 28], and *k*-Dop [29].

In this paper, we do experiment based on a liver model. Considering the complexity of liver model's irregularity, the complexity of structuring the bounding box, and the difficulty of the bounding box update after deformation, we use the following collision detection algorithm.

We use a three-dimensional point representing the top of the surgical instrument. We can obtain the position of the virtual instrument by tactile delivery engine in real time, then calculating the distance between the static particle of liver model and surgical instrument successively. When the distance is less than zero, we identify that the collision happened, then returning the vertex sequence number of the particle in collision.

### 4.5. Procedure of Deformation Simulation

The method simulating deformation of soft tissue and calculating the feedback force using the SPH method based on dynamic local interaction area in detail is as follows:

(1) set up the meshless particle model and initialize each variable as zero;
(2) build the deformation equations in terms of Vogit viscoelastic model;
(3) manipulate the force feedback device to stress soft tissue model with external force *f*_ext_, and return the serial number of contact particle after collision detection; at this time, the internal force *F*_in_(*t*) = 0, *t* = 0;
(4) calculate each particle as follows:
  (a) calculate the relative distance Dis between the particle and the contact point;
  (b) if Dis is less than Dr, calculate the particle's acceleration, strain, and stress by SPH method precisely; if Dis is between Dr and Tr1, multiply the contact particle's acceleration by weighting factor of the first transmission layer directly to get the particle's acceleration; if Dis is between Tr1 and Tr2, multiply the contact particle's acceleration by weighting factor of the second transmission layer directly; if Dis is larger than Tr2, the particle's acceleration will be set zero;
  (c) calculate the particle's velocity and distance in terms of Newton's Second Law:
    (22) $$\begin{array}{c} v_{i}^{t+1}=v_{i}^{t}+\Delta ta_{i}\left(t\right),\\ x_{i}^{t+1}=x_{i}^{t}+\Delta tv_{i}^{t+1}; \end{array}$$
  (d) draw the state of each particle on the display dynamically using OpenGL functions;
(5) calculate the out force of contact particle:
  (23) $$\begin{array}{c} f_{out}^{i}=f_{ext}+\sigma_{\alpha \beta }^{i}; \end{array}$$
(6) output the force through the feedback device, so the user can feel the tactile feedback with soft tissue.

In step (b), we need to calculate the particle's acceleration, strain, and stress by SPH method precisely. The main procedure is as follows.

(i) For current particle *p*_*i*_, search its neighbor particles *p*_*j*_ within smooth radius *h* in the list; then calculate the smooth nuclear function equation (9) between the current particle and its neighboring particles in support domain.
(ii) Calculate *ρ*_*i*_ of the particle by (15).
(iii) Calculate acceleration *a*_*i*_(*t*):
  (24) $$\begin{array}{c} a_{i}\left(t\right)=-\frac{dv_{i}\left(t\right)}{dt}+\frac{\sigma_{i}\left(t\right)}{m_{i}}+\frac{F_{ext}^{i}}{m_{i}}, \end{array}$$
 where *F*_ext_^*i*^ is sum of external force at *p*_*i*_, *σ*_*i*_ is the internal force, that is, stress at *p*_*i*_, *m*_*i*_  is mass of particle *p*_*i*_, and *dv*_*i*_(*t*)/*dt* can be calculated by (14).
(iv) Calculate the displacement: disp_*i*_(*t*) = *x*_*i*_(*t*) − *x*_*i*_(*t*_0_).
(v) Calculate *ε*_*αβ*_^*i*^ by previous displacement and state equation (16).
(vi) Record each particle's current volume strain *ε*_*kk*_^′*i*^ and shape distortion *e*_*αβ*_^′*i*^, and then use (2) to calculate the new volume strain and shape distortion by previous strain.
(vii) Calculate each particle's volumetric stress *σ*_*kk*_^*i*^ and deviatoric stress *S*_*αβ*_^*i*^ by (3).
(viii) Calculate stress state *σ*_*αβ*_^*i*^ of each particle by (1).

## 5. Experiment

**Figure 5:**
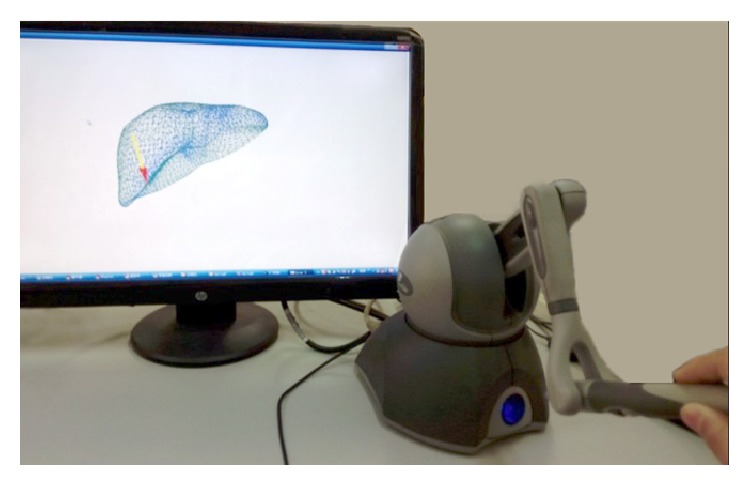
Experimental environment based on PHANTOM Omni.

In this paper, all experiments were implemented in the same environment, specific as follows: Window XP operating system, AMD Phenom(tm) II X2 3.0 GHz PC with 2.0 GB RAM, Visual Studio 2005, and Open Graphics Library. The haptic feedback device we used is PHANTOM OMNI developed by SensAble Company in USA. It provides a complete API which is fully compatible with the OpenGL API and simplifies the haptic rendering thread. The experimental environment based on PHANTOM Omni is shown in Figure 5.

**Figure 6:**
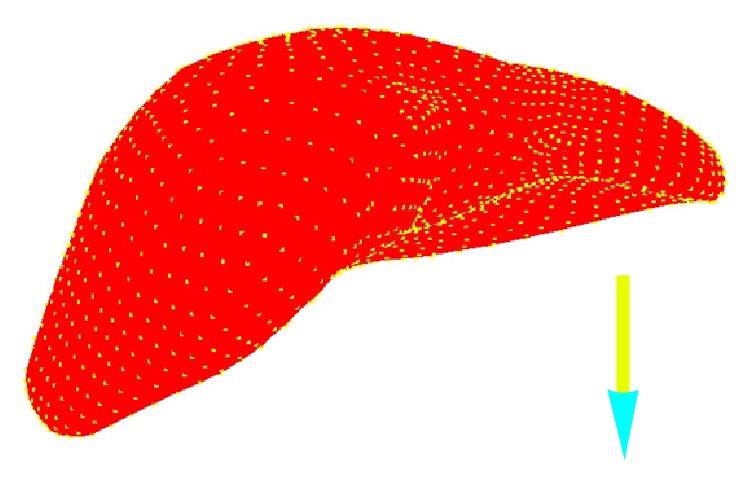
The initial state of liver model.

**Figure 7:**
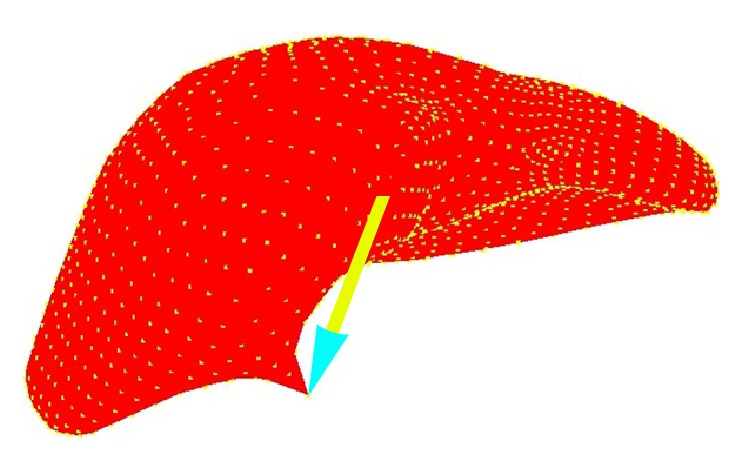
Liver deformation under pull force.

**Figure 8:**
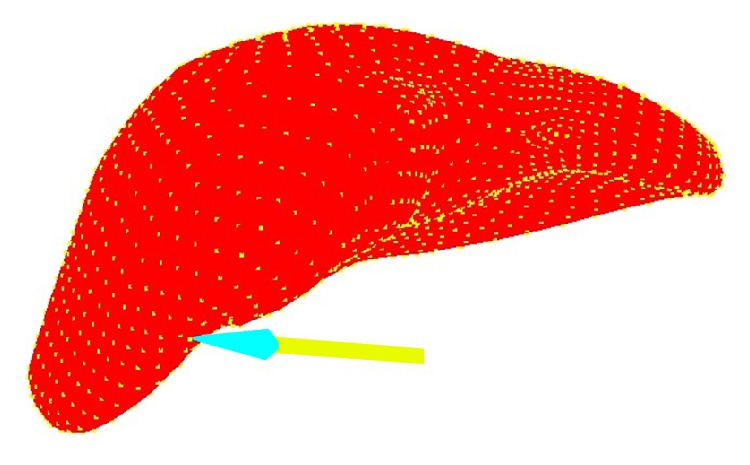
Liver deformation under press force.

We performed simulations of manipulation of a liver model in order to demonstrate the feasibility of the procedure with more realistic organ geometries. The model geometry was obtained from segmented CT data. The initial state of the liver is shown in Figure 6 in which the pencil is representative of the arm of PHANTOM OMNI. The liver is sampled into uniformly distributed 3690 particles carrying the same mass and density. When pull force is applied to the upper part of the liver body, a snapshot of deformation process is shown in Figure 7. When press force is applied to the upper part of the liver body, a snapshot of deformation process is shown in Figure 8.

**Figure 9:**
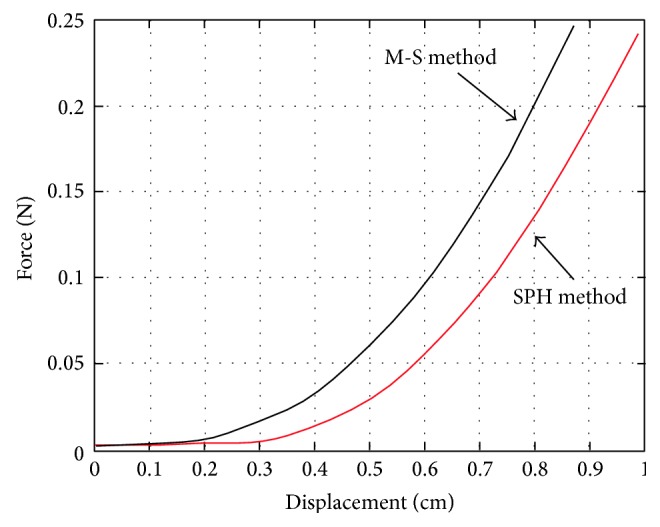
The force-displacement curve with M-S model and SPH method.

**Figure 10:**
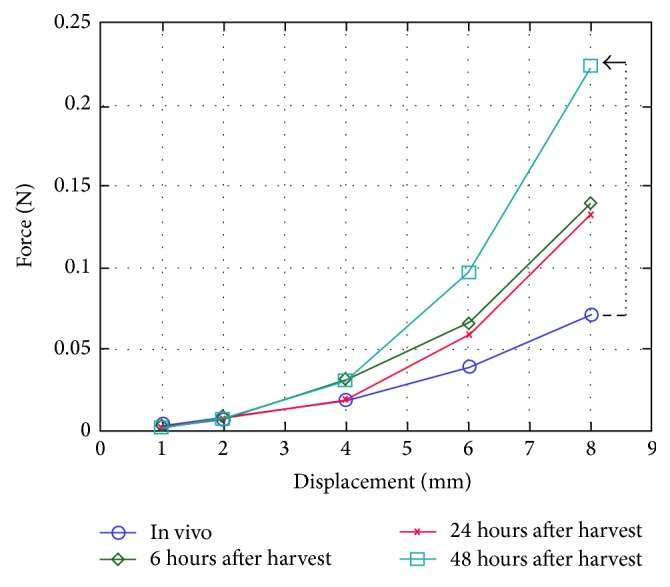
Force-displacement curve based on pig liver in vitro and ex vivo [30].

To verify the accuracy of SPH, we implemented the simulations based on M-S and SPH separately. We can get the displacement of the same node in the model through applying load on liver model with virtual surgery instrument. After fitting them with quadratic curve, we can find they are coherent overall, as shown in Figure 9. De et al. [30] did experiments based on the pig liver in vivo and ex vivo and presented the steady-state force response of pig liver, as shown in Figure 10. Comparing Figure 9 with Figure 10, the results indicate that SPH methodology is suitable for simulating the liver tissue deformation and force feedback calculation.

In this paper, we simulate three liver models of different quantity of particles using mass-spring model and global SPH, respectively; the frame rates of them are indicated in Table 1. Compared to M-S model, SPH method has a heavy computational load and poorer real time though it has higher precision. In virtual surgery simulation, stable tactile feedback required the refreshment rate no less than 300 HZ. The result in Table 1 shows that SPH method does not suit to calculate the feedback force when the number of particles is larger. While the number of particles is around 1300, it could basically achieve a smooth and stable tactile feedback.

**Figure 11:**
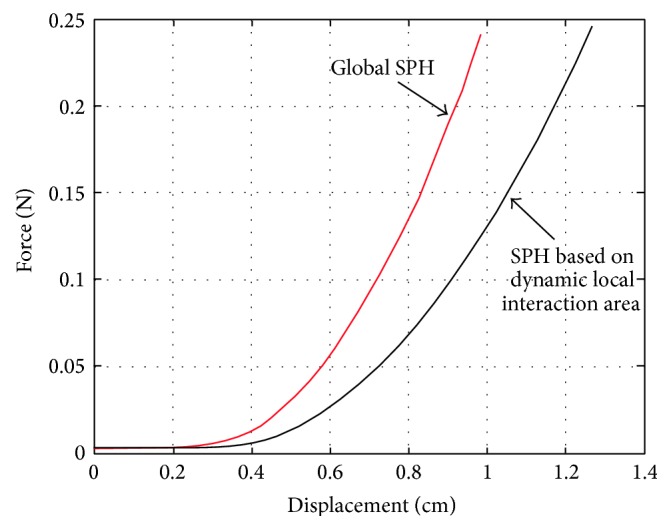
Force-displacement curve of two SPH methods.

We implemented the deformation and force feedback using global SPH and SPH based on dynamic local interaction area separately. Applying the same load with same direction and value on the same node in the model, we can get the force-displacement curve, as shown in Figure 11. As can be seen from the figure, the overall trend of the two curves is basically consistent, and the interaction force using SPH based on dynamic local interaction area is smaller than the global SPH method since only part of the domain is discredited and the number of particles involved in the calculation is reduced which result in a local error.

In order to verify the time real performance of SPH based on dynamic local interaction area, we simulated the deformation of five liver models with different numbers of particles using global SPH method and SPH based on dynamic local interaction area, respectively. Since the value of the smooth radius in the SPH method affects the number of particles involved in the calculation directly which would impact on the computational efficiency, we census the running time of different smooth radiuses. The running time of global SPH is shown in Table 2, and that of SPH based on dynamic local interaction area is shown in Table 3, in which the unit of running time is second.

The following conclusions can be drawn from Tables 2 and 3.

(1) The running time of SPH based on dynamic local interaction area and global SPH method improves with the increase in the number of particles, but SPH based on dynamic local interaction area has a higher computational efficiency significantly.
(2) The more particles the model contains, the more effective the SPH based on dynamic local interaction area is. However, the computational efficiency has few gaps for the model containing a small number of particles.
(3) The value of smooth radius *h* has impacted on the computational efficiency directly; while the smooth radius becomes greater, the computational efficiency of process will become lower and timeliness is worse.
(4) If the model contains a small number of particles, the value of smooth radius impacts on the running time slightly. This is due to the small number of particles distributed sparsely. Setting different smooth radius has little effect on the number of particles involved in calculation. So it almost does not influence the running time of program.

Refreshment rate of stable tactile feedback should be more than 300 HZ in virtual surgery simulation, so the computing time should be less than 0.0033 s. Comparison of Tables 2 and 3 shows that when smooth radius is set to smaller values such as 0.3, the global SPH would be difficult to meet the requirements of the haptic frame rate with the number of particles over 1289. But SPH based dynamic local interaction area could satisfy tactile frame rate while the number of particles increases to 3670. It greatly improves the simulation in real time compared with global SPH.

The real time performance of SPH based on local dynamic interaction area is related not only with the smooth radius, but also with the interaction region and spread layers. Now we discuss the impact of the different transmission layers and interaction radius to the real time performance of algorithm.

When smooth radius *h* is 0.6 and transmission layer is 2, the influence of different interaction area radius on the real time performance of algorithm is shown in Table 4. When smooth radius *h* is 0.6 and *k*_1_ = 0.5*∗f*_ext_, the influence of different layers on the real time performance is shown in Table 5, in which *N* indicates the propagation layer. It could be concluded from Tables 4 and 5 that as the length of interaction area radius and the number of propagation layers increase, the real time of algorithm becomes worse, and the value of the radius had a greater influence on real time performance. The reason is that when the interaction area radius becomes larger or spread layers increase, the number of particles involved in the calculation will get more which results in an increase in the computational cost. Moreover, the calculation in interaction area is more complex than that in spread region. Therefore, the value of interaction radius has a greater effect on the real time performance.

## 6. Conclusions

In this paper, we adopts Voigt viscoelastic mechanical model to present the characteristics of biomechanics which has strongly physical realism. In the meanwhile we use SPH method as a meshfree technique to solve the deformation process and feedback force which enhances the accuracy of simulation compared with approaches based on meshes.

However, SPH method has a heavy computational load and poorer real time though it has higher precision. In order to improve the computational efficiency, we proposed a SPH method based on dynamic local interaction area. We divide the model into deformation area and nondeformation area and assume that the deformation is only affected by nodes in the deformation area, and the nodes in nondeformation area will remain still. Deformation area contains two parts, namely, interaction area and transmission area. Interaction area is the region greatly influenced by the surgical instruments, in which the SPH method is adopted to do accurate calculation of particles; transmission area is the effective propagation range of force, in which the physical quantities of all particles are obtained by decaying physical quantities of the force points. The particles in different transmission layers have different effect on the deformation of the model. So we set weighting factors to indicate the influence degrees of different transmission layers.

Experimental results show that SPH methodology is suitable for simulating the liver tissue deformation and force feedback calculation, and SPH based on dynamic local interaction area has a higher computational efficiency significantly compared with usual SPH. SPH based on dynamic local interaction area can ensure 300 HZ frame rate when the number of particles is under certain number which satisfies the need of smooth haptic feedback in virtual surgery. Because the accuracy of SPH method also depends on the arrangement of the particles, when the force loads on some particles in disordered arrangement or topological structure, the result will be unstable. So our next tasks mainly focus on the following.

1. Improving the stability of SPH method: during the experiments, particles shocked irregularly when the time step was set to large. In the next work, we will study the factors for stability of SPH, such as smooth radius, kernel function, and the distribution of particles.
2. Verifying the accuracy of force feedback: real medical surgery is very strict and does not allow any deviation. Therefore, it must validate the accuracy of force feedback in virtual surgery and make a comprehensive evaluation for authentication methods, for example, joining the deformation time, deformation range, feelings of trainers, and the test of physicians into the evaluation program.

## Figures and Tables

**Table 1:** Computational efficiency of M-S model and SPH method.

Number of particles	M-S model		SPH method	
	Time (s)	Rate (HZ)	Time (s)	Rate (HZ)
3670	0.00064	1562.50	0.026	38.7
1289	0.00037	2702.70	0.003	333.3
289	0.00007	14285.7	0.00018	5555.6

**Table 2:** Running time of different models based on global SPH.

Number of particles	Running time			
	*h*			
	*h* = 0.3	*h* = 0.5	*h* = 0.6	*h* = 0.8
3670	0.026	0.089	0.15	0.31
1289	0.003	0.011	0.018	0.04
743	0.0012	0.0054	0.0069	0.12
572	0.0007	0.0022	0.0035	0.0073
289	0.00047	0.00056	0.00063	0.0011

**Table 3:** Running time of different models by SPH based on dynamic local interaction area.

Number of particles	Running time			
	*h*			
	*h* = 0.3	*h* = 0.5	*h* = 0.6	*h* = 0.8
3670	0.0032	0.0056	0.0079	0.02
1289	0.001	0.0014	0.0021	0.0056
743	0.00063	0.00073	0.00097	0.0017
572	0.00047	0.00052	0.00055	0.0012
289	0.00031	0.00033	0.00033	0.0004

**Table 4:** Calculation efficiency with different local radius.

Interaction radius	*k*_1_ = 0.5∗*f*_*ext*_	*k*_1_ = 0.8∗*f*_*ext*_	*k*_1_ = 1.0∗*f*_*ext*_
Time (s)	0.0027	0.0073	0.02
Rate (HZ)	370.4	137.0	50

**Table 5:** Calculation efficiency with different transmission layer.

Transmission layer	*N* = 1	*N* = 2	*N* = 3	*N* = 4
Time (s)	0.00186	0.0027	0.00357	0.00473
Rate (HZ)	537.6	370.4	280.1	211.4
